# Additive Manufacturing of Polypropylene: A Screening Design of Experiment Using Laser-Based Powder Bed Fusion

**DOI:** 10.3390/polym10121293

**Published:** 2018-11-22

**Authors:** Iñigo Flores Ituarte, Olli Wiikinkoski, Anton Jansson

**Affiliations:** 1 Department of Materials and Production, Section of Sustainable Production, Faculty of Engineering and Science, Aalborg University, Copenhagen 2450, Denmark; 2 Department of Mechanical Engineering, School of Engineering, Aalto University, Espoo 11000, Finland; olli.wiikinkoski@aalto.fi; 3 Department of Mechanical Engineering, Örebro University, Örebro 70182, Sweden; Anton.Jansson@oru.se

**Keywords:** additive manufacturing, powder-bed fusion, laser sintering, polypropylene, process parameter optimization, mechanical properties, computer tomography

## Abstract

The use of commodity polymers such as polypropylene (PP) is key to open new market segments and applications for the additive manufacturing industry. Technologies such as powder-bed fusion (PBF) can process PP powder; however, much is still to learn concerning process parameters for reliable manufacturing. This study focusses in the process–property relationships of PP using laser-based PBF. The research presents an overview of the intrinsic and the extrinsic characteristic of a commercial PP powder as well as fabrication of tensile specimens with varying process parameters to characterize tensile, elongation at break, and porosity properties. The impact of key process parameters, such as power and scanning speed, are systematically modified in a controlled design of experiment. The results were compared to the existing body of knowledge; the outcome is to present a process window and optimal process parameters for industrial use of PP. The computer tomography data revealed a highly porous structure inside specimens ranging between 8.46% and 10.08%, with porosity concentrated in the interlayer planes in the build direction. The results of the design of experiment for this commercial material show a narrow window of 0.122 ≥ *Ev* ≥ 0.138 J/mm^3^ led to increased mechanical properties while maintaining geometrical stability.

## 1. Introduction

Additive manufacturing (AM) of thermoplastic polymers using powder-bed fusion (PBF) has become an alternative to conventional manufacturing methods [1]. Equipment manufacturers integrate AM systems to their manufacturing processes [2] as PBF technology offers many advantages, particularly for the production of geometrically complex components in low- to mid-volume batches [3,4]. In addition, technological projections consider AM an important element of future digitalization of manufacturing [5]. Hypothetically, AM will co-exist and, in certain cases, replace conventional manufacturing [6]. The adoption of AM contributes to reducing the upfront cost linked to conventional manufacturing, simultaneously improving flexibility in manufacturing new products [7]. A paradigm change involves mass-production needing to become highly flexible to answer individualized needs in a resource-friendly manner [8]. 

Accordingly, AM can solve the so-called scale-scope dilemma, as the feasibility of product variety and, therefore, mass-customization is without cost penalties [9]. However, the implementation of polymer PBF is limited to few materials. The market shows that 90% of complete industrial consumption is limited to polyamide 12 (PA12) or blends, for example dry blends of glass-filled, aluminum-filled, and carbon-fiber-filled polyamides. The remaining is distributed among other polymer powders, such as PA11, TPU, PEBA, and PEEK [10]. In addition, every kilogram of PBF powder sold corresponds to approximately 200 tons of conventional polymeric material sold at the same time [11]; thus the real significance of polymer PBF in manufacturing remains minor.

New materials are crucial to opening new market segments and applications. Material options are limited due to the complexity of the PBF process [12], which involves multiple machine and process variables including machine design, laser, process parameters, recoating, and heating. Simultaneously, these variables are intertwined with intrinsic and extrinsic powder material characteristics involving thermal, optical, rheological, particle size distribution, and powder morphology [11]. The interaction phenomenon between laser and material is complex and understanding polymer chemistry and process parameters interaction is difficult to master. Furthermore, the secret recipe of process variables for new commercial materials are kept as trade secret, thus slowing down any competition.

The processing of PP by AM presents extra difficulties as the material presents a strong tendency to shrink and warp due to a high degree of crystallinity [13]. This manuscript contributes to increasing the understanding of the PP sintering process and providing systematic information to understand process–property relationships. The study’s first research objective is (1) to investigate the PBF of a commercially available PP powder including both intrinsic and extrinsic characteristics and key process parameters in relation to the achievable mechanical properties. In addition, an original study on porosity of PBF processed PP is introduced. The second objective is (2) to compare these results with the existing body of knowledge through a literature research. Finally, this research (3) defines a process parameter window for PP sintering and provides guidelines for its use as an alternative to conventional materials in laser-based industrial PBF. 

To highlight some of the results of this research, sintered PP specimens showed a highly porous structure, similar to other PBF materials [14]. The porosity is concentrated in the interlayer planes in the build direction. The study revealed that increasing energy density allows obtaining a higher tensile strength at the cost of decreasing the elongation at break. The results of the design of experiment shows a narrow window of 0.122 ≥ *Ev* ≥ 0.138 J/mm^3^ for this commercial PP powder that led to improved mechanical properties. 

## 2. Materials and Methods 

### 2.1. Open Hardware and Software PBF Machine

**Figure 1:**
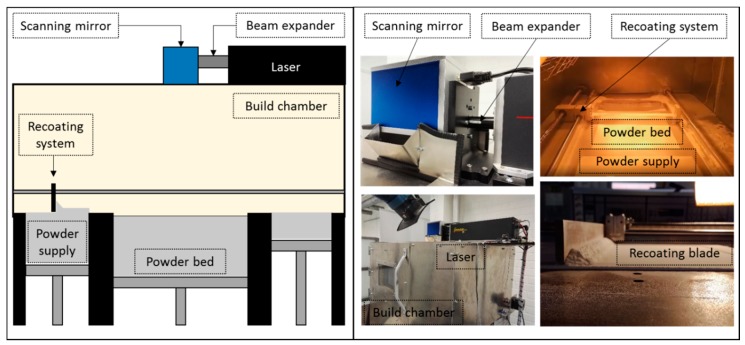
Open hardware and software PBF system.

This research used an open software and a hardware PBF platform, designed and constructed for polymer-material testing and process-development activities (see Figure 1). The machine’s design and architecture is based on commercial PBF equipment. The machine uses a 100 W CO_2_ laser Synrad ti-100w, (Synrad, Garching, Germany) equipped with a galvanometer Sinogalvo model SG8216 (Sinogalvo, Beijing, China) with F-theta lenses (Wavelength-tech, Singapore) with a focal length (EFL) of 573.2 mm and a theoretical center spot size (1/e^2^) of 930 μm. Typical machine process variables include layer thickness, recoating speed and powder-bed temperature. 

These variables are fully controlled with a custom-made user interface that drives micro-stepper motors for layer thickness control and recoating operations, along with IR heating lamps IR heaters Master hall 1500 (Master Climate Solutions, Pastrengo, Italy) to build chamber temperature control. Accordingly, IR sensors (Melexis, Ypres, France) and thermocouples (RKC Instrument Inc., Tokyo, Japan) are used to control and maintain a steady powder-bed temperature. The laser-galvanometer process variables are controlled using RepliSLS3D open software [15] allowing us to modify and control process variables, such as laser power, scanning speed, scanning patterns, laser compensations, contour, and hatching strategies.

### 2.2. Extrinsic Properties: Morphology and Particle Size Distribution

This study’s test material was a mechanically mixed Coathylene^®^ sint polypropylene (PP) (Axalta Polymer Powders, Bulle, Switzerland) and fumed nano-silica (SiO_2_) composite (Axalta Polymer Powders, Bulle, Switzerland). The neat PP was provided by Advanc3d materials; and the material supplier recommended mixing the neat PP with at least 0.25 wt % Nano-SiO_2_. 

**Figure 2:**
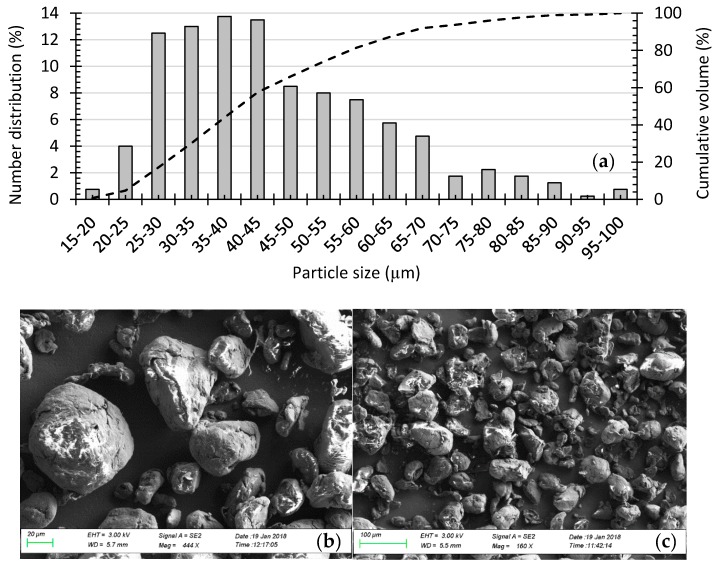
Powder morphology and extrinsic properties. (**a**) Particle-size distribution of coathylene polypropylene; (**b**) A SEM picture with a magnitude of 444× and 20 µm scale and (**c**) SEM picture with a magnitude of 160× and 100 µm scale.

This additive is typically used to improve flowability in recoating operations [16]. Additionally, the additive avoids electrostatic behavior of semi-crystalline thermoplastic powders, which negatively impacts both powder flowability and packing density during recoating operations [17]. To ensure homogenous mixing of the compound powder, the material remained for 30 min in a rotating blender, at 30-rpm revolution speed. The analysis of particle-size distribution used a scanning electron microscope (SEM) Zeiss Sigma VP (Jena, Germany), with image analysis using ImageJ open software [18]. Figure 2 illustrates both morphology and particle-size distribution of neat PP.

The thermoplastic powder has an apparent density of 0.905 g/cm^3^. The average particle size is approximately 45 µm (i.e., 0–25 µm, 4.75%; 25–45 µm, 52.75%; 45–80 µm, 38.5%; +80 µm, 4%). Particle-size analysis revealed the powder has a cumulative-size distribution of 30.29 µm (D10), 49.01 µm (D50), and 76.63 µm (D90). A right-skewed distribution is revealed by the histogram, with nearly 90% of the particles lower than 60 µm. The SEM images show the powder is not completely spherical, with particles having varied morphologies from nearly spherical to an elongated ‘potato’ shape. The powder, however, proved suitable for processing with PBF, especially after mixing with the nano-SiO_2_. Such mixing had a dramatic impact on improving powder-bed quality and powder flowability during recoating operations [16].

### 2.3. Intrinsic Properties: Melting and Crystallization Characteristics

**Figure 3:**
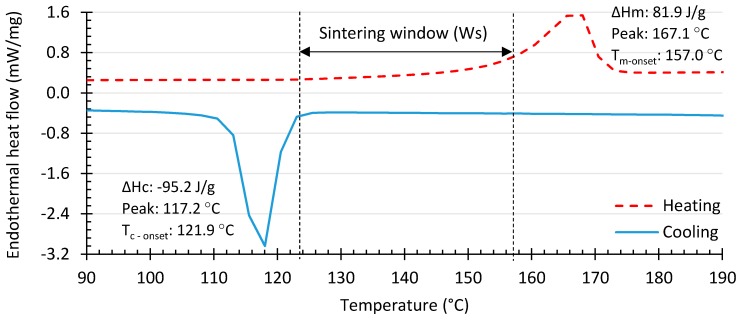
DSC thermograph for the neat polypropylene.

A differential scanning calorimeter (DSC), Netzsch DSC 204 F1 (Malmö, Sweden) was used to determine the sintering window (W_s_) for the PP. The sintering window is the difference between the melting- (*T*_m_-onset) and initial crystallization (*T*_c_-onset) temperatures [19]. The heating temperature range was set between −20 °C and 220 °C, and the cooling range was between 220 °C and room temperature. The heating and cooling rates were set at 10 °C/min. Figure 3 displays the results, which illustrate the material has an approximate sintering window of 35.1 °C, with a melting peak of 167.1 °C; we determined a degree of crystallinity of 39.58%.

Research shows a high degree of crystallinity usually results in higher shrinkage and lower ductility, but also lower porosity and better tensile properties [20]. Subsequently, the processing temperature must be precisely controlled between the melting and crystallization temperatures to prevent early crystallization. The DSC result shows a wide sintering window of the studied PP, therefore accommodating small variations in the optimum processing temperature and temperature gradients during the PBF process.

To minimize thermal gradients around the sintered area, the bed temperature was set close to the melting temperature. Thus, geometrical deviations are reduced from both nominal and undesirable effects, such as warpage, shrinkage, and curling effects [21]. In our experiment, we set the part bed temperature at 150 °C, which is above the onset of crystalline melting point determined from the DSC data, 17.1 °C lower than the material’s melting peak. 

### 2.4. Design of Experiments (DOE), Control Variables, and Measurements

Some of the most important parameters involved in polymer PBF process are: laser power (*P*), laser scan speed (*V*), laser scan spacing or hatch distance (*S*), layer thickness (*Lt*), and part bed temperature (*T*_b_) [12]. The relation between some of these variables is given by the energy density (*Ev*), defined as the relative applied laser energy per volume of material. This relation is often used to describe the correlation between key process variables in PBF and is calculated by Equation (1) for the SLS process [22]. 

(1) $$\;Ev\;\left(\frac{J}{mm^{3}}\right)=\;\frac{P\;}{V\;.Lt\;.S}\;$$

Factor *V* determines the exposure time of the CO_2_ laser beam working on the powder. At a fixed laser *P*, a higher *V* implies shorter exposure; therefore, less energy is transferred to the layer, resulting in a lower degree of melting and penetration [23]. In this study, two of the most influential process parameters involving *P* and *V*, were altered at discrete intervals, while the other parameters were set as constant (i.e., *T*_b_, *Lt*, *S*, Nano-SiO_2_ content, build orientation, scanning pattern, and contour scanning). Research shows the significant effect of these parameters on the overall performance of both the PBF process [23] and mechanical properties. Table 1 displays the experimental levels of the DOE and a detailed list of process parameters and constant parameters.

Interactions between the process and constant parameters have a non-linear and complex effect on control parameters, such as porosity, part density, mechanical strength, elongation at break, and others [24]. Nevertheless, by varying parameters in a controlled manner, modelling and stabilizing the process is possible. These procedures enable defining a suitable process parameters window for PBF [25]. Subsequently, we performed a full factorial DOE to correlate the process parameter and energy density with the control variables. The experimental work evaluated the impact of process parameters on the following control variables: (1) ultimate tensile strength, (2) elongation at break, and (3) porosity. During the mechanical testing, three tensile specimens were manufactured for each experimental combination thus 27 tensile specimens. The specimens followed the standard test method for tensile properties of plastics ASTM D638-02a (type IV). In performing the porosity evaluation and computer tomography (CT) measurements, the gripping area of the same tensile specimens were used to produce rectangular specimens. The specimens had an average volume of 418.56 mm^3^ and dimensions of 10.36 × 10.33 × 3.9 mm^3^.

Measurement of the tensile strength and elongation at break of the produced specimens used MTS Insight 30 kN electromechanical testing systems and MTS 634.12F-24 extensometer, the clamping of the specimens was performed with the MTS Advantage wedge action grips. Regarding the porosity measurements, all the 27 rectangular specimens, previously cut from the tensile bars, were scanned using a SkyScan 1272 system from Bruker (Bruker MicroCT, Kontich, Belgium). The samples were scanned three at a time using an acceleration voltage of 50 kV and a filament current of 200 µA. The resulting volumes had an isometric voxel size of 5 µm. Investigation of volumes used the commercially available software VG studio MAX. Performing porosity calculations in VG studio used a region of interest inside the bulk of the material. 

## 3. Results and Discussion

### 3.1. Mechanical Properties and Porosity Evaluation of Sintered Polypropylene

Tensile testing of the 27 specimens produced stress–strain curves until fracture. The data reveal the appearance of strain hardening during the stretch processing until the eventual occurrence of fracture at an average tensile strength of 19.9 MPa and average elongation at break of 2.9%. After several experiments with the PP material, an initial process window was established by trial and error. The upper and lower *Ev* limits in this DOE were 0.100 J/mm^3^ and 0.150 J/mm^3^, respectively. We found that higher *Ev* values caused warping, curling, and geometrical distortion, thereby preventing the production of usable specimens within the required geometrical tolerance level according to the ASTM standard. In addition, higher energy levels caused build failure due to the contact of the recoating blade with the curled sintered layers in the powder bed. Conversely, lower *Ev* values showed a clear lack of fusion between layers, thus producing unusable specimens for material testing.

Overall, the result of our DOE shows brittle part behavior with values under 9% for elongation at break, and values under 34 MPa for tensile strength properties. These properties are typically achieved by injection molding processes of polypropylene [26]. Nevertheless, the mechanical performance falls within an expected range, especially regarding the existing body of knowledge (see Table 2) for the PBF process of commercial PP powders [27,28]. 

**Figure 4:**
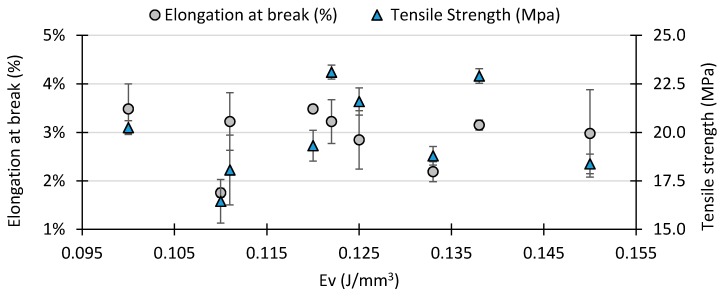
Variation of the tensile strength and the elongation at break with respect to energy density.

The results of the tensile testing including tensile strength and elongation at break as a function of the *Ev* are shown in Figure 4, at the same time the standard error of means in displayed for each experimental point. The tensile strength and elongation at break data set shows how these two-control variables present two opposing trends, revealing the strength-ductility trade-off dilemma. At a fixed laser power, a higher scan speed implies shorter exposure time between the laser and powder, consequently transferring less energy to the powder bed. This results in lower cohesion between layers, which is associated with reduced tensile strength. In overall, when energy density is at its highest level, a higher tensile strength can be obtained at the cost of decreasing the elongation at break. Conversely, when energy densities are at a lower level, the elongation at break is maximized at the cost of decreasing tensile strength.

**Figure 5:**
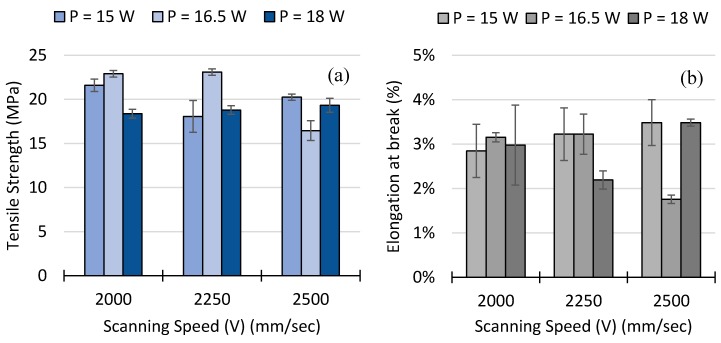
Variation of (**a**) the tensile strength and (**b**) elongation at break as a function of laser power (*P*) and scan speed (*V*).

The tensile strength and elongation at break of the specimens built with different processing parameter combinations are shown in Figure 5, at the same time the standard error of means in displayed for each experimental combination. The graph on the left-hand side illustrates that at fixed 15 W the tensile strength decreases and then increases, at 16.5 W the tensile strength increases and then decreases, and at 18 W the tensile strength consistently increases with increasing scan speed. At the same time, the graph on the right-hand side b shows that at 15 W the elongation at break increases with higher scan speed, at 16.5 W the elongation at break increases and then significantly decreases and at 18 W the elongation at break first decreases and then increases with higher scan speed. In summary, the interaction between power and scanning speed reveals a non-linear phenomenon that cannot be explained in monotonic trends.

In summary, the maximum tensile strength found was 23.1 MPa at an energy density of 0.122 J/mm^3^ with a process parameter combination of *P* = 17 W and *V* = 2250 mm/s. However, the maximum elongation at break found was 3.5% at 0.100 J/mm^3^ with a process parameter combination of *P* = 15 W and *V* = 2500 mm/s, the lowest energy density tested in this DOE. 

Comparing these results with existing research in PBF for PP, [24,25] confirm the tensile strength initially increases as the energy density increases. However, after the upper limit is reached, the tensile strength drops slightly to reach a plateau. The presented DOE was incapable of replicating this phenomenon, as the range energy density was limited to a narrower window due to excessive geometrical distortion. Nevertheless, the selected range of *Ev* from 0.100 J/mm^3^ to 0.150 J/mm^3^ could produce tensile specimens with the required dimensional accuracy according to the standard.

**Figure 6:**
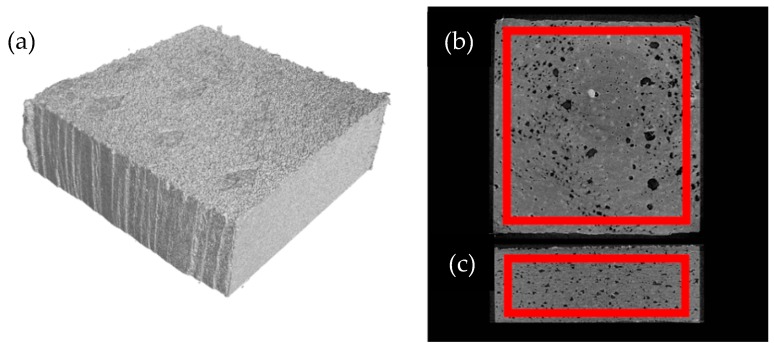
(**a**) 3D rendering of a sample fabricated with *Ev* = 0.111 J/mm^3^; (**b**) A slice from the interlayer region in the sample; (**c**) A slice from the side of the sample. The red square indicates the region of interest used to calculate porosity.

**Figure 7:**
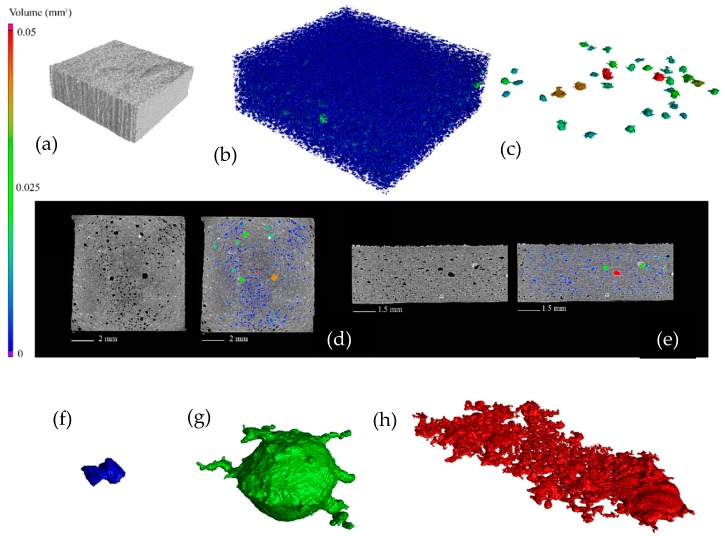
(**a**) 3D rendering of a sample fabricated with 0.110 J/mm^3^; (**b**) total internal porosity; (**c**) only large defects; (**d**) interlayer slice with/without color-coded porosity; (**e**) side-view of porosity; (**f**) small, acute, pores; (**g**) spherical pores; (**h**) layered porosity.

As mentioned in the method section, the porosity calculations were carried out in each specimen in a region of interest. The region captures the bulk properties of the samples; an example of this region can be seen in Figure 6. This figure shows both the 3D rendering of the sample and an interlayer top- and cross-section view of a specimen. The red square indicates the region of interest used to calculate porosity. Figure 7 shows the CT analysis of the same specimen that corresponds to manufacturing *Ev* of 0.110 J/mm^3^ and process parameters on *P* = 16.5 W and *V* = 2500 mm/s.

**Figure 8:**
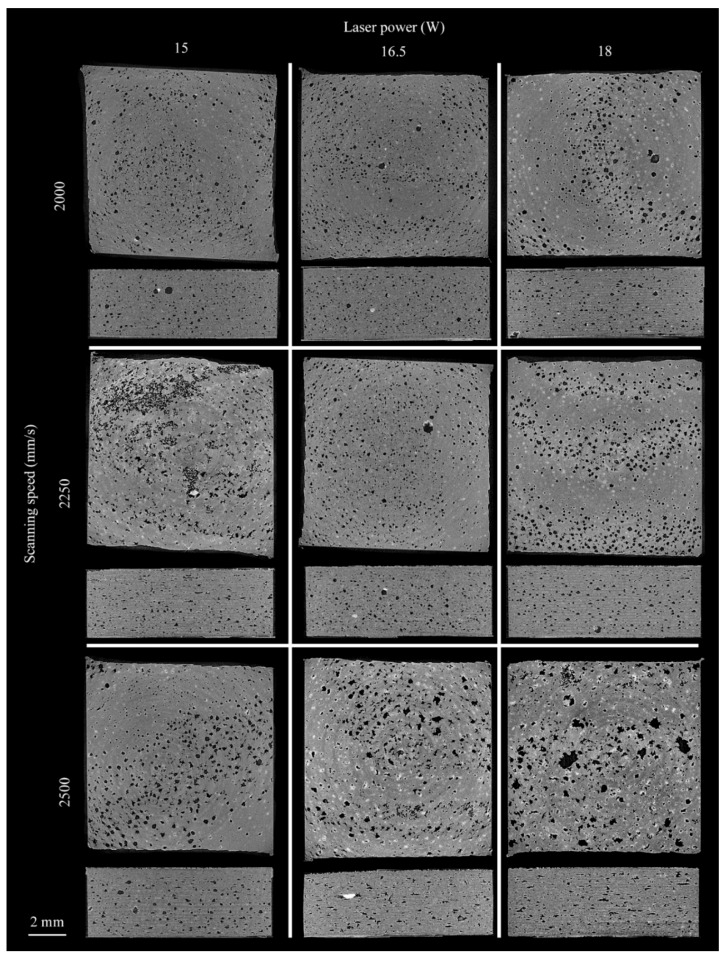
Representative slices from each of the sample batches. The slices display the interlayer connection and the side view of the samples. The sample slices are organized as: **top row**: 2000 mm/s scanning speed; **middle row**: 2250 mm/s scanning speed; **bottom row**: 2500 mm/s scanning speed; **left column**: 15 W laser power; **middle column**: 16.5 W laser power; **right column**: 18 W laser power.

**Figure 9:**
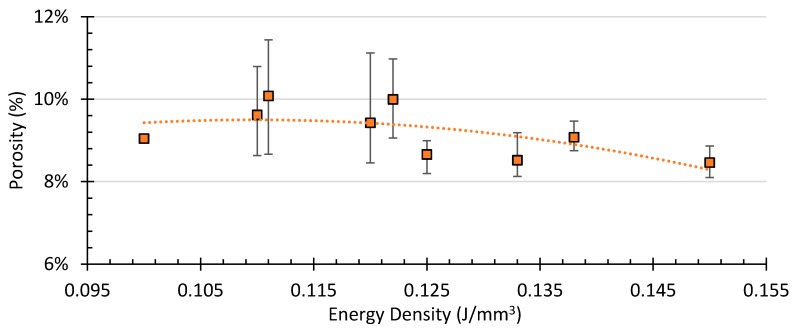
Porosity in the samples as a function of energy density and a polynomial trend-line.

The CT data revealed a highly porous structure inside specimens ranging between 8.46% and 10.08%, with porosity concentrated in the interlayer planes, these porosity results are comparable to previous studies on porosity of material processed using laser based PBF [29]. Figure 7a–e illustrate the porosity and poor fusion of the layers in the build direction. From the overall assessment of the CT images, the larger porosity defects allowed classification into three categories: small sharp, spherical, and layered. Figure 7f–h illustrates these three main categories of defects. The small defect displayed in Figure 7f has a volume of 0.00045 mm^3^; small defects comprise the bulk of porosity in all samples in this case defect is from a sample fabricated with 0.133 J/mm^3^. Large spherical defects often stretch their diameter through several build layers; the defect displayed in Figure 7g has a volume of 0.025 mm^3^ and a radius of approximately 200 µm. In this case, the large spherical defect corresponds to a sample fabricated with 0.110 J/mm^3^. Layered defects comprise many small defects connected along build layers. The defect displayed in Figure 7h stretches for approximately 3 mm and has a volume of 0.16 mm^3^. In this case, the example of a layered defect corresponds to a sample fabricated with 0.120 J/mm^3^. Figure 8 is a visual representative example of the porosity as a function of the process parameters *P* and *V*. Overall, significant differences in porosity between samples from the same batches appeared, as some samples contained layered defects, while others in the same batch did not. Fully developed layered porosity was consistently found in at least one specimen for the four lowest energy densities (0.100–0.120 J/mm^3^). In the case of higher *Ev*, the trend in Figure 9 shows how the average porosity decreased slightly as energy density increased.

**Figure 10:**
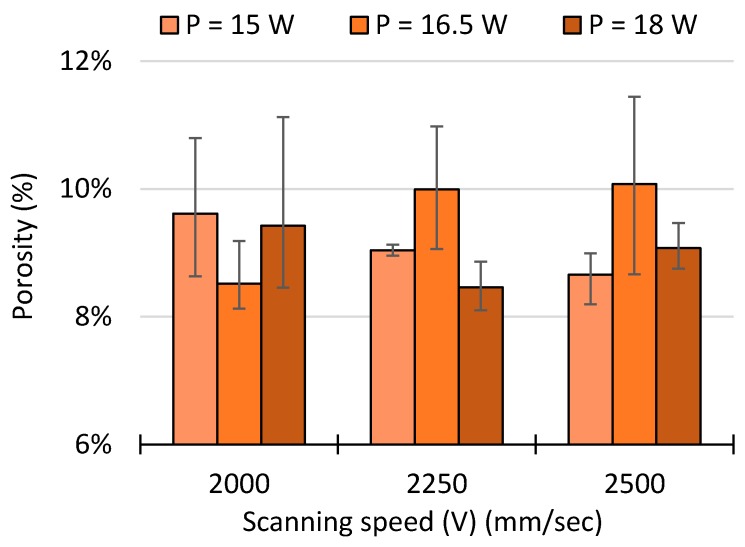
Variation of the porosity percentage as a function of the laser power (*P*) and scan speed (*V*).

The energy density and process parameters strongly influence the microstructure and porosity levels of the sintered parts. All specimens contained open and closed porosities; Figure 10 shows how the degree of porosity is dependent on process parameters. At fixed 15 W the porosity first increases and then decreases, at 16.5 W the tensile strength increases and then decreases, and at 18 W the tensile strength consistently increases with higher scan speeds. At a fixed laser power, higher scan speed implies lower energy levels, and therefore larger porosity levels. 

### 3.2. Main Effect Plots, ANOVA, and Contour Plot for Optimum Process Parameters

**Figure 11:**
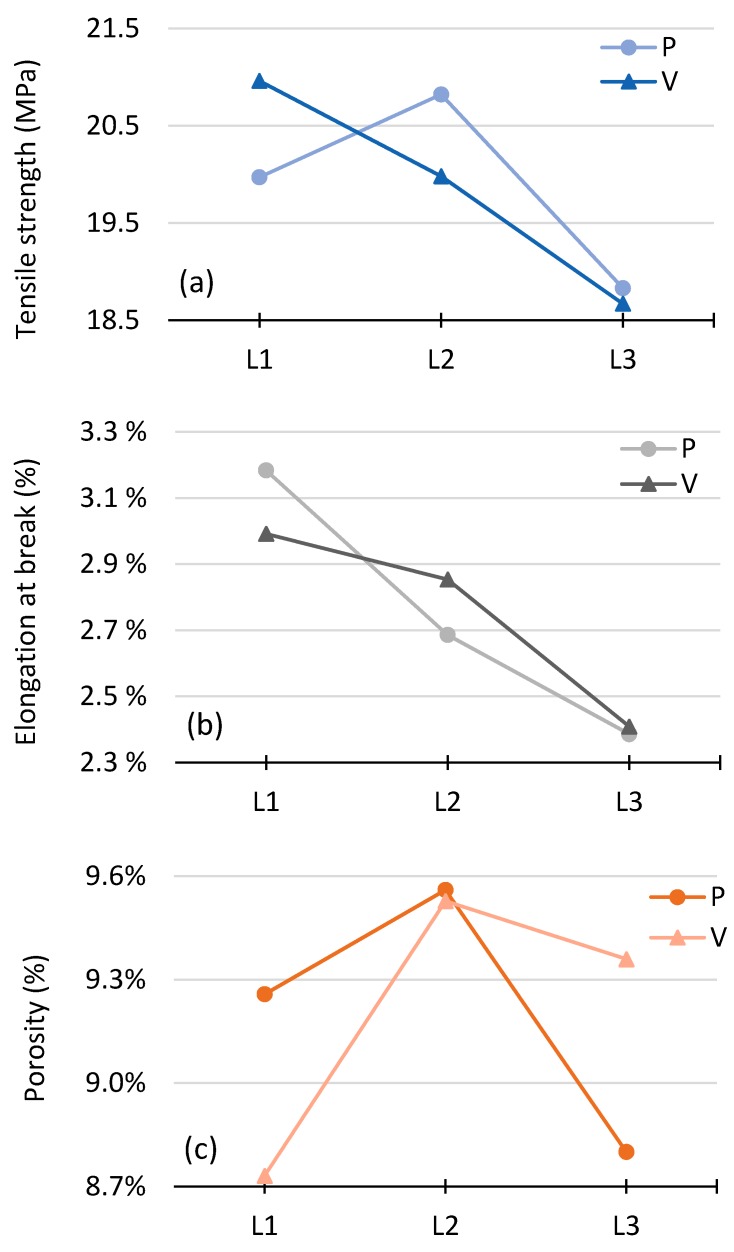
Main effect plot for means of (**a**) tensile strength; (**b**) elongation at break; and (**c**) porosity.

Figure 11 presents the calculated means per level for the effect of process parameters on tensile strengths, elongation at break, and porosity. The higher the difference between the minimum and maximum value of the mean, the higher the effect, and the higher the subsequent influence of the process variable over the response. In this regard, *V* is statistically more significant than *P* for tensile strength, whereas *P* is statistically more significant than *V* for elongation at break. However, the data reveals little difference between the significance of *P* and *V*; thus, both process parameters have a similar effect. All three figures strongly interact between *P* and *V*; therefore, the interaction between them and second-order effects are significant to the achievable tensile strength, elongation at break and porosity.

As part of the design of experiment (DOE), an analysis of variance (ANOVA) test is conducted with a confidence level of 95% (α = 0.05). The ANOVA test included both independent variables *P* and *V*. The ANOVA included all first order, second order, and interaction terms. The statistical significance of the terms can be assessed by looking at the test’s *P*-value. The more the *P*-value approaches zero the more significant is the effect of the term over the response [30]. 

Table 2 shows the result of the full ANOVA including first order, second order, and interaction terms. However, due to the limited sample size (*n* = 27) the results of the ANOVA failed to construct a strong regression model to estimate tensile strength, elongation, and porosity. The experimental results show that measures of the strength of the relationship, such as R-Squared varied substantially, with an R-square of 27.92%, 30.68%, and 26.44% for tensile strength, elongation, and porosity, respectively.

Although the low R-squared indicates high-variability in the experimental results, the *P*-Value can be still use to study the significance of process parameters *P* and *V*. The interpretations of the significant variables are the same for both high and low R-squared models [30]. As a consequence, for tensile strength the effect of first order and second order term of *P* has more statistical significance when compared with *V*. Whereas, the effect of first order and second order terms of *V* have more significance when compared to *P*. The effect of the interaction term between *P* and *V* show the strongest significance for elongation at break in comparison to tensile strength and porosity respectively.

**Figure 12:**
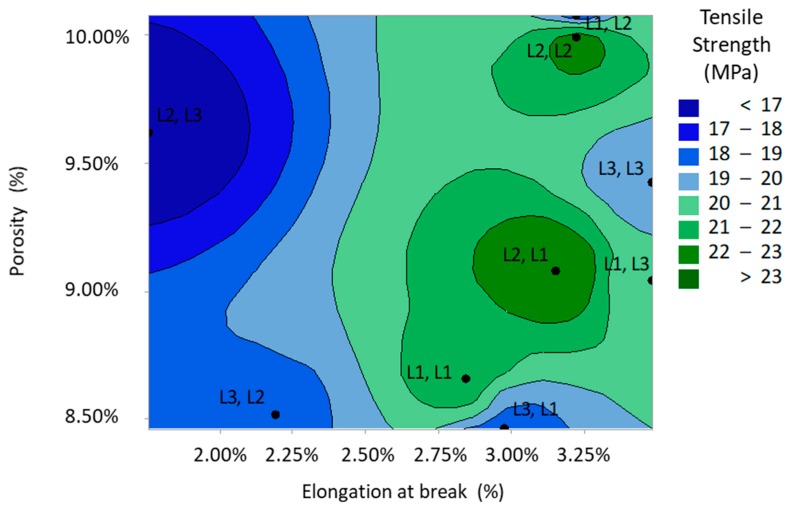
Contour plot of tensile strength (MPa) versus porosity (%) and elongation at break (%).

To define the optimal set of process parameters that led to increased mechanical properties, Figure 12 shows the contour plot for all nine tested experimental combinations. The diagram uses a distance method for interpolation, which is fit for surfaces with isolated extreme values, sampling is not intensive enough and might have a large variability as it is the case of this DOE. In this regard, the color map from blue to green, corresponds to achievable tensile strength. The horizontal axis of the contour plot corresponds to elongation at break and the vertical axis represents the porosity level. The combination of process parameters is also displayed in the contour plot.

The contour plot shows that the use of high power (L3) is detrimental to tensile strength, the main effect plot in Figure 11 shows the same effect. In summary, two of the solutions led to highest tensile strength on average while maintaining a similar level of elongation at break. In summary, *P*(L2) = 16.5 W and *V*(L2) = 2250 mm/s as well as *P*(L2) = 16.5 W and *V*(L1) = 2000 mm/s led to the highest tensile strength of 23.1 MPa and 22.9 MPa and elongation at break of 3.22% and 3.15%, respectively. On the contrary, the porosity seems to decrease with increasing energy density; and therefore, *P*(L2) = 16.5 W and *V*(L1) =2000 mm/s was able to produce 90.93% dense parts.

### 3.3. Process Parameters Window for Commercial PP Powders

To contextualize our research, Table 3 shows a comparison of our experimental results with the existing body of knowledge for PBF of PP, although the methods, materials, and process, along with control variables and parameters, differ in each study. The results of material intrinsic and extrinsic properties and a process parameter window is presented for five commercially PP materials and experimental results with blends (i.e., Coathylene PP+PA12, PP-R201 Trial Corp., iCoPP, PPCP22, and Rolaserit PP). 

Bearing in mind that processing conditions (e.g., PBF machine and process parameters), material chemistry and composition, and control variables and measurement systems differ from experiment to experiment, obtaining general conclusions is possible. Comparing the intrinsic material characteristics, such as the sintering window (Ws) and the powder-bed temperature (*T*_b_), the DSC presents similar results from experiment to experiment. The average recommended *T*_b_ is 146 °C (stdev: 14.21) and the commercially available PP blends show a Ws of 32.05 °C (stdev: 7.55). Although not all the included research presents a detailed analysis of the extrinsic properties, the results are still consistent with D50 = 60.87 µm (stdev: 10.75) and D90 = 91.3 µm (stdev: 20.8). Regarding the flowability linked to extrinsic properties of the powders, none of the compared research reported issues with the powder flowability. Nevertheless, this is a factor that clearly affects the performance of new polymer materials on PBF [16], and its wider adoption within AM service providers [14].

Typical layer thickness (*Lt*) for processing PP is within the range of 100 µm to 150 µm. On the other hand, key process parameters, such as power and scanning speed, differ from study to study. This difference relates to the different type of energy sources (i.e., laser and heating) equipped in the PBF machines. Nevertheless, the compilation presented Table 3 shows a range of energy densities (*Ev*) capable of processing PP with consistent mechanical properties and dimensional stability. Subsequently, the reported average minimum, maximum, and optimal *Ev* values are 0.118 J/mm^3^ (stdev: 0.038), 0.375 J/mm^3^ (stdev: 0.181), and 0.209 J/mm^3^ (stdev: 0.089) respectively. 

Overall, this study shows that although commercially available PP is an alternative material in PBF, the mechanical properties are inferior to parts made by conventional methods, such as injection molding or other PBF friendly polyamides, such as PA12. On average, we can use the reference data of 9% for elongation at break and 34 MPa for tensile strength properties, which is typically achieved by injection molding of polypropylene. Consequently, the average tensile strength and elongation at break achieved is 21.33 MPa (stdev: 4.16) and 7.65% (stdev: 5.85). Nevertheless, the benefit of utilizing PP can be justified by both its potential for cost reduction compared to typical PBF polymers, such as PA12, and for diversification of material opinions in polymer PBF.

## 4. Conclusions

Commodity thermoplastic polymers, such as polypropylene and its blends, are required to expand new material capabilities of existing laser-based PBF systems. This initial screening DOE research shows that both a relatively high part density and good mechanical properties can be achieved in two ways: by studying the impact of energy density, and by defining a suitable process parameters window for semi-crystalline polypropylene. This article contributes to elucidating some of the complex relations between intrinsic and extrinsic polymer features, processing parameters conditions, and the achievable mechanical properties and porosity. 

Consequently, this research presents both the intrinsic and extrinsic properties, such as melting and crystallization characteristics, particle size distribution and mechanical of a commercial PP, and the impact of key process parameters. In the presented study, power (*P*) and scanning speed (*V*) were systematically modified in a controlled full-factorial screening design of experiment (DOE). The results of the DOE are classified according to the process parameters as a function of the achievable tensile strength, elongation at break, and porosity. 

In this study, tensile specimens were fabricated using an open hardware and software PBF system. Using a section of the gripping area for each tensile specimen, we studied porosity using a CT scanner. Comparing the results of this work to the existing body of knowledge and its outcome creates a process windows for PP commercial powders by PBF. According to the results of this work, the following conclusions can be made:

- Overall, the result of our DOE on the commercial polypropylene material shows average mechanical properties of 19.9 MPa for ultimate tensile strength, 2.9% for elongation at break, and 9.21% of porosity.
- The CT data revealed a highly porous structure inside specimens ranging between 8.46% and 10.08%, with porosity concentrated in the interlayer planes in the build direction.
- The study revealed that increasing energy density allows obtaining a higher tensile strength at the cost of decreasing the elongation at break. Simultaneously, porosity is also reduced at higher energy densities. However, the phenomena reveals a strong interaction between process variables; and therefore, a non-linear behavior.
- The material under study required a narrow energy density window to allow producing dimensionally stable parts. The experiment shows that, a narrow range of constituent parameters led to optimal results. In our experiment, the *Ev* values fell between 0.100 J/mm^3^ to 0.150 J/mm^3^. Higher energy densities caused warping, curling, and geometrical distortion. Conversely, lower *Ev* values showed a clear lack of fusion between layers, thus producing unusable parts.
- Based on the DOE results, a narrow window of 0.122 ≥ *Ev* ≥ 0.138 J/mm^3^ led to maximum tensile strength as well as increased elongation at break. In this regard, a *P* = 16.5 W and 2000 ≥ *V* ≥ 2250 mm/s led to the best results.

The results of this research show the process parameters that led to highest possible achievable mechanical properties and lower porosity levels. Future research will include a larger sample size to avoid an analysis susceptible to outliers resulting from measurement errors as well as to improve the reproducibility and significance of the study. Larger sample sets (typically 40 or more) should be used to be able to construct a precise regression model. Furthermore, future research should investigate the impact of additional process parameters involving the effect of layer thickness, build orientation, and laser-scanning strategies to improve the mechanical properties of commercially available AM polypropylene. Additionally, the use of other reinforcing fibers and additives is certainly of interest to limit the shrinkage and warping effect during the sintering process as well as to tolerate higher *Ev* values to improve further mechanical properties of sintered polypropylene.

## Figures and Tables

**Table 1:** Factors and levels for the full factorial DOE

**Varying Process Parameters**				
Factors	Units	Level (L1)	Level (L2)	Level (L3)
Laser power (*P*)	(W)	15	16.5	18
Scanning speed (*V*)	(mm/s)	2000	2250	2500
**Constant Process Parameters**				
Bed temperature (*T*_b_)	(°C)	150		
Layer thickness (*Lt*)	(mm)	0.150		
Laser scan spacing or hatch distance (*S*)	(mm)	0.4		
Nano-SiO_2_	(wt %)	0.25		
Build orientation	-	Horizontal to the build platform		
Contour scanning (*C*_s_)	(W)	*C*_s_ = PLevel-2.5		
Scanning pattern: (Dash line) *C*_s_: (Dash point line)	-	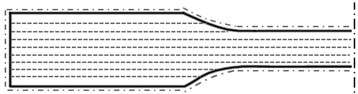		

**Table 2:** Full ANOVA table of the mechanical properties for first order, second order, and interaction terms.

		Tensile Strength		Elongation at Break		Porosity	
Source	DF	Adj SS	*P*-Value	Adj SS	*P*-Value	Adj SS	*P*-Value
Regression	5	45,582	0.197	0.000657	0.145	6.0653	0.229
*P*	1	7466	0.261	0.000001	0.892	1.1805	0.239
*V*	1	0.297	0.82	0.000075	0.314	0.8574	0.313
*P* ^2^	1	12,124	0.156	0.000006	0.776	1.6945	0.161
*V* ^2^	1	0.175	0.862	0.000014	0.659	1.3989	0.201
*P* × *V*	1	3927	0.412	0.000198	0.109	0.2508	0.582
Error	21	117,706		0.001486		16.8722	
Lack-of-Fit	3	78,671	0	0.00025	0.333	2.8274	0.335
Pure Error	18	39,035		0.001235		14.0448	
Total	26	163,289		0.002143		22.9375	

**Table 3:** Literature research for process parameters window and mechanical performance for PBF of polypropylene specimens

Ref.	[31]	[32]	[33]	[34]	[24,25]	[24,25]	DOE
**Material**	Coathylene (80%) + PA12 (20%)	PP-R201 Trial Corp. (Japan)	iCoPP	PPCP22 (Diamond Plastics)	Rolaserit PP (ROWAK)	Rolaserit PP (ROWAK)	Coathylene + SiO_2_ (0.25 wt %)
**Machine**	DTM Sinterstation 2000	HRPS-IV SLS system	DTM Sinterstation 2000	DTM Sinterstation 2000	EOS Formiga P100	DTM Sinterstation 2500	Open PBF system
***T*** **_b_** **(°C)**	160	150	122	148	N/A	N/A	150
**W_s_ (°C)**	38	21 *	N/A	34.1	27.3	27.3	35.1
**Particle Size (µm)**	N/A	D10 (38.1), D50 (63.6), D90 (106)	N/A	D50 (70)	N/A	N/A	D10 (30.29), D50 (49.01), D90 (76.63)
***P*** **(W)**	6, 7, 8, 9	8.25, 11, 13.75, 16.5	15, 20, 25	15, 20, 25	18, 21.5, 25	21, 41	15, 16.5, 18
***Lt*** **(mm)**	0.1	0.15	0.1	0.1	0.06, 0.1, 0.12 *	0.1, 0.3 *	0.15
***V*** **(mm/s)**	1257	1500, 2000, 2500, 3000	5080	3500, 5080	4000, 5000	5000	2000, 2250, 2500
***S*** **(mm)**	0.15, 0.25 *	0.2	0.23	0.23	0.15, 0.2, 0.25	0.2	0.3
**Min. *Ev* (J/mm^3^)**	0.191	0.092	0.128	0.128	0.120	0.070	0.100
**Max. *Ev* (J/mm^3^)**	0.477	0.367	0.214	0.311	0.694	0.410	0.150
**Opt. *Ev*** **(J/mm^3^)**	0.191 *	0.306 *	N/A	N/A	0.25 *	0.25 *	0.138
**Max. TS (MPa)**	29	19.9	19,5	N/A	18	18.5	23.1
**Max. EAB (%)**	4	N/A	N/A	N/A	13	15	3.5

* The values were not provided directly, and they have been interpreted from experimental data. [24,25,31,32,34].
